# Deep Learning Encoding for Rapid Sequence Identification on Microbiome Data

**DOI:** 10.3389/fbinf.2022.871256

**Published:** 2022-06-24

**Authors:** Jacob Borgman, Karen Stark, Jeremy Carson, Loren Hauser

**Affiliations:** Department of Data Science, Digital Infuzion, Inc., Gaithersburg, MD, United States

**Keywords:** deep learning, microbiome, convolutional neural networks, rapid sequence identification, encoding, embedding, denoising

## Abstract

We present a novel approach for rapidly identifying sequences that leverages the representational power of Deep Learning techniques and is applied to the analysis of microbiome data. The method involves the creation of a latent sequence space, training a convolutional neural network to rapidly identify sequences by mapping them into that space, and we leverage the novel encoded latent space for denoising to correct sequencing errors. Using mock bacterial communities of known composition, we show that this approach achieves single nucleotide resolution, generating results for sequence identification and abundance estimation that match the best available microbiome algorithms in terms of accuracy while vastly increasing the speed of accurate processing. We further show the ability of this approach to support phenotypic prediction at the sample level on an experimental data set for which the ground truth for sequence identities and abundances is unknown, but the expected phenotypes of the samples are definitive. Moreover, this approach offers a potential solution for the analysis of data from other types of experiments that currently rely on computationally intensive sequence identification.

## Introduction

The identification of known sequences and of new variants related to known sequences has been foundational to biological science over decades. The original Smith-Waterman algorithm (Smith and Waterman, 1981) identified the most optimal alignments between sequences but was computationally demanding and therefore slow. BLAST was first introduced in 1990 (Altschul et al., 1990) as a more rapid approximation and has evolved to its current form (Camacho et al., 2009) as the main workhorse for sequence identification. The use of k-mers (Edgar, 2004) has also become a widely used method for faster rapid approximations based on string searches and counts. Because of the large numbers of reads found in many experimental microbiome samples and the frequency with which bacteria contain multiple copies of the 16S gene many times with single-base variation, there is a need for a solution that can further reduce computational demands on sequence identification while simultaneously providing single-base resolution of sequence variation. Moreover, improved methodology for identification of which single-base variants in a microbiome sample represent sequencing errors and which are likely to be true biological sequence variants would assist in obtaining accurate abundance results.

A search of PubMed using the term “Microbiome” generates over 100,000 listings and a graph showing exponential growth over the last 10 years. The Human Microbiome Project (https://portal.hmpdacc.org/), which contains just 18 microbiome studies, contains over 30,000 samples. Most published microbiome studies contain small number of samples, and therefore, their statistical resolving power is low. In order to increase the resolving power of studies on a specific subject, larger studies containing many thousands of samples are desirable, and the capability to combine multiple studies for meta-analysis would be useful. In either case, this means that thousands of samples would need to be processed with speed and accuracy using a single set of analysis tools. Reduction in the computational burdens of using these tools would promote the ability of more researchers to conduct studies with larger samples.

The output of commonly used microbiome tools falls into two general categories: operational taxon units (OTUs) that group together closely related strains into higher level taxonomic units and amplicon sequence variants (ASVs) that strive to achieve the base pair level accuracy required to bring taxonomic identification to the strain level. The microbiome analysis pipeline tool QIIME2 (Bokulich et al., 2018) uses the widely adopted VSEARCH algorithm (Rognes et al., 2016) for microbiome data analysis at the OTU level, while also permitting optional selection of a broader range of algorithms. VSEARCH uses a k-mer-based approach to speed sequence identification and error resolution, originally inspired by USEARCH (Edgar, 2010). USEARCH evolved to include UPARSE (Edgar, 2013) for OTU analysis using default 97% identity for clustering. Many clustering methods including mothur-average (Schloss et al., 2009), UPARSE, and UCLUST (Edgar, 2010) are benchmarked and compared in Kopylova et al. (2016) and applied to test microbiota data sets at the OTU level.

OTU methods intentionally speed analysis by settling for higher level taxonomic resolution and that is frequently sufficient for phenotypic studies. ASV methods take on the additional challenge of trying to achieve finer-grained taxonomic resolution by distinguishing sequence variation that is due to errors in the sequencing process from true biological sequence variants. Among ASV methods, the DADA2 microbiome analysis tool (Callahan et al., 2016) uses a probabilistic model to identify amplicon sequence variants (ASVs) with high sequence fidelity and has been chosen by multiple comparative studies as having the highest biological resolution for differentiating closely related and/or low abundance strains (Nearing et al., 2018; Caruso et al., 2019; Prodan et al., 2020). The UNOISE3 algorithm (Edgar, 2016) uses a kmer-based approach to sequence identification and error correction to produce ASVs. UCLUST, UPARSE, VSEARCH, and UNOISE3 allow for pooling all samples or clustering sequence reads for each sample individually for error correction. DADA2 uses a subset of samples to learn its error profile and then applies this error model to one sample at a time. The Deblur algorithm (Amir et al., 2017) operates on each sample separately for clustering to identify ASVs. Caruso et al. (2019) found DADA2 and UNOISE to be preferable for maximizing detection of true community members but note Deblur may be more appropriate for minimizing detection of spurious ASVs. UNOISE has been shown to have significantly higher speed (Nearing et al., 2018) than DADA2. Performance benchmarks and detailed comparison of the algorithmic similarities and differences among the VSEARCH, DADA2 and UNOISE3 algorithms is given in Tremblay and Yergeau (2019) and among DADA2, UNOISE3 and Deblur in Nearing et al. (2018).

Microbiomics is an ideal field for applying recent advances in machine learning that may offer speed advantages in combination with high accuracy when there is sufficient training data available. There is a large quantity of publicly shared microbiome data, with countless studies revealing the pivotal role microbial populations play in establishing and maintaining healthy conditions within diverse set of ecosystems, including the human body. The gut microbiome alone has been implicated in bone and brain development, obesity, diabetes, autoimmune conditions, autism, cardiovascular disease, metabolic disorders, inflammatory bowel disorders, and drug response (Cho and Blaser, 2012; Jandhyala et al., 2015; Levy et al., 2017; Thursby and Juge, 2017; Barlow et al., 2018; Gilbert et al., 2018). The presence or absence of certain bacterial populations are often directly linked to these medical conditions. Effective tools for characterizing healthy versus unhealthy microbial populations with resolution as close to the strain level as possible have an important impact on biological discovery, potentially leading to new diagnostics and treatments. Soil and plant microbiomes are also subjects of active research, where the same tools can be applied to determine microbial composition and lead to valuable interventions.

Unprecedented levels of accuracy in other fields have been achieved by the expansion of machine learning through the development of Deep Learning algorithms. In 2012, the convolutional network AlexNet created a sensation with its dramatic improvement demonstrated in an established computer vision competition using the ImageNet challenge data (Krizhevsky et al., 2012). Since then, image based neural networks have continued to evolve, both in terms of architecture and training strategies, from recurrent neural networks to the now widely applied Transformer (Vaswani et al., 2017) design. Aside from computer vision, these algorithms have revolutionized other important areas such as speech and text recognition and have created headline news with vast AI improvements in specialized domains such as board games [e.g., AlphaGo (Silver et al., 2016)] and protein folding [AlphaFold (Jumper et al., 2021)].

Deep learning algorithms have been applied to classify the phenotype of microbiome and metagenome samples. Asgari et al. (2018) showed that deep learning can outperform random forest classifiers and support vector machines for phenotypic prediction from 16S data when the number of samples is large. Zhao et al. (2021) use kmer embeddings and convolutional neural networks, recurrent neural networks, and attention mechanisms to predict taxonomic classifications and sample-associated attributes of whole microbiome data at the level of a read. They use additional methods such as voting to determine the phenotype of each sample from the deep-learning-predicted phenotype of the reads. This enables the predictor to consider many thousands of read sequences and it achieves accuracy at phenotypic prediction comparable to existing methods. An early application of deep recursive neural networks to metagenomic data did not show much improvement over other methods for metagenomic classification but the ability to learn hierarchical representations of a data set that is produced could be useful (Ditzler et al., 2015). Furusawa et al. (2021) chose an unusual approach to perform image analysis on Gram-stained fecal samples to classify their microbiome state with a deep convolutional neural network. Although the prediction success was low for fecal state, particularly on samples from adjacent time points, it had more success in predicting quantitative changes in microbial abundances. García-Jiménez et al. (2021) explored the creation of deep latent spaces for prediction of the ecological composition of a microbiome sample using minimal sequencing features and incorporating sample environmental metadata such as rainfall and plant age. These methods are generally not intended to produce exact microbial composition based on rigorous sequence variant identification or optimal abundance estimates at the level of ASVs. They focus more on the ability to accurately categorize the sample as a whole with respect to a relevant phenotype (i.e., a population characteristic of interest). However, for a deeper understanding of the microbial population, the population dynamics and the ability to approach the mechanisms by which the microbiome exerts its influence, an accurate analysis of its composition at the true sequence variation level provides more scientific insight than a phenotypic classifier.

Sequence identification in the closely related field of metagenomics is an area where deep learning algorithms are beginning to be applied. The Seeker tool (Auslander et al., 2020) addresses the challenging problem of detecting bacteriophage in metagenomic sequence data since bacteriophage evolve rapidly, quickly losing sequence similarity to known bacteriophage. It uses Long Short-Term Memory (LSTM) networks, a type of Recurrent Neural Network (RNN), trained on bacteriophage and bacteria sequences to detect subtle differences in sequence usage and is able to predict which sequences in the metagenome are bacteriophage and which are from bacteria, even when homology to known bacteriophage is very low. Virfinder2 (Ren et al., 2020) is a convolutional neural network (CNN) that learned to predict viral sequences by training on the differences between prokaryotic and viral DNA sequences. Its success does not rely on known sequence homologies or use of pre-defined features such as kmers. While using more traditional machine learning and not deep learning, the VirSorter2 algorithm (Guo et al., 2021) has demonstrated considerable success in identifying both RNA and DNA viruses within metagenomic samples. It relies on a collection of known viral motifs and annotations that are used as input features to a set of random forest classifiers each trained on a major viral group. Its modular design allows for easy updates as known viral diversity grows. While each of these tools is successful at sequence analysis and appropriate for metagenomics, their algorithmic approaches are not readily adapted for microbiome analysis that relies on 16S amplicon sequences from a single bacterial gene and then attempts to identify the population of bacteria represented by those sequences.

Herein, we describe a deep learning approach to finding ASVs and obtaining their abundance estimates on sample sequencing data obtained from mock communities of known bacterial composition. We will show that using a latent sequence space based on all known bacterial V4 sequences from the 16S gene and using a Convolutional Neural Network algorithm trained to map V4 sequences obtained from experimental data into this space will match or better the accuracy of the best available open source microbiome tools in significantly shorter computational time. A denoising method that starts with clustering of experimental sequence data in the V4 16S latent sequence space achieves accurate abundance estimates. Although motivated by the desire for improved sensitivity and accurate abundance estimation of the microbial community, we demonstrate that the output still supports phenotypic prediction by comparison to previously published results for four data sets where the exact microbial composition is unknown, but the phenotypes of the samples are unambiguous.

This approach may be extensible to other types of experimental sequence data in addition to microbiome where single nucleotide resolution, correction of likely sequencing errors and accurate abundance estimation are desirable. We refer to this mathematical approach as Deep Learning Encoding for Rapid Sequence Identification (DERSI).

## Materials and Methods

In order to identify a method for both rapid and accurate identification of ASVs from microbiome experiments that use 16S sequence, a series of steps were performed. The steps were used on data from microbiome sequence analysis using the V4 region of the 16S gene.

**FIGURE 1:**
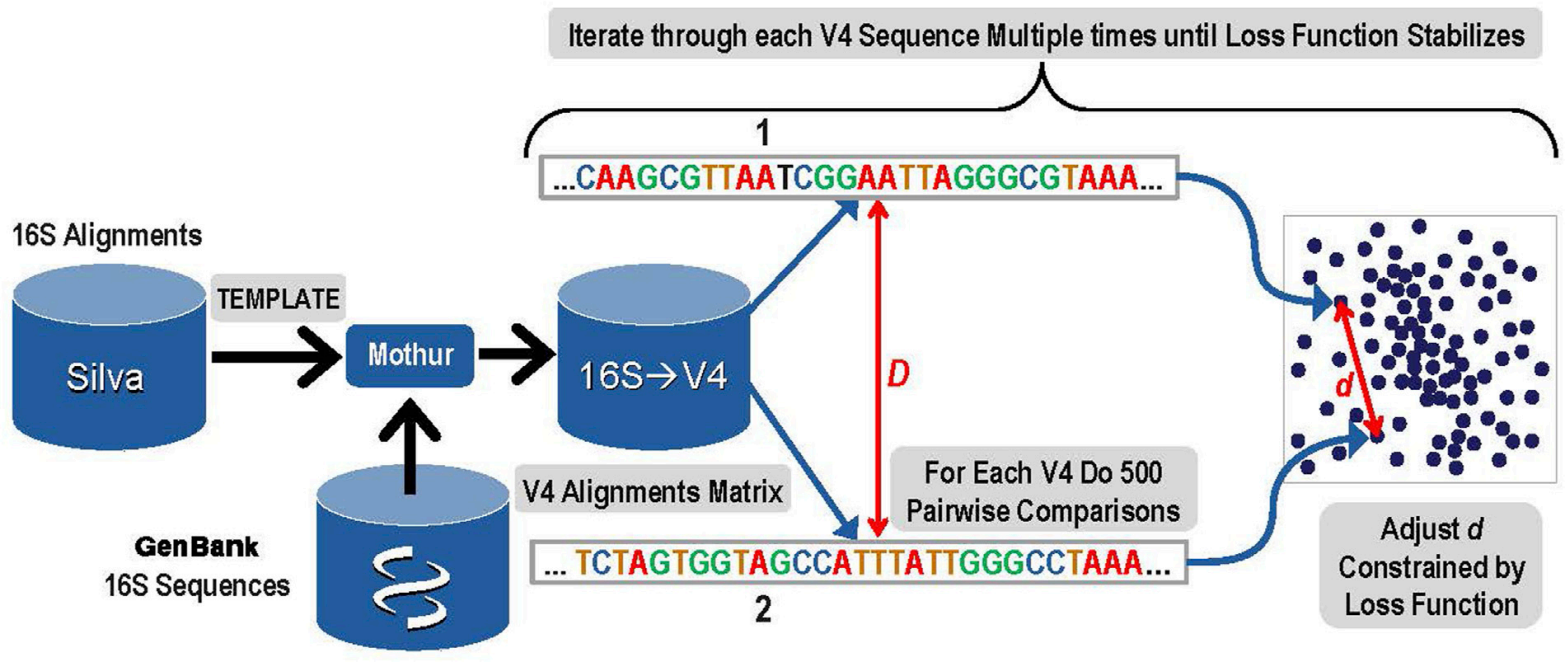
Overview of Step 1: The construction of a latent space that represents known V4 sequences and their alignment distances with reduced dimensionality while maintaining a high degree of accuracy.

1. The first step was to create a 10-dimensional latent space that encoded the distances among all known bacterial and archaeal V4 sequences. An overview of this step is presented in Figure 1. A copy of the Silva rRNA database (Yilmaz et al., 2014) version 132 that contained alignments for known 16S sequences was supplemented with sequences from GenBank. The Silva database consisted of a set of more than 200,000 samples of known 16S sequences placed into alignment with each other. Due to gaps and insertions, ∼50,000 possible nucleic acid positions are present in this alignment matrix for the full-length 16S gene. The alignment matrix exhibits extreme sparsity and any manipulation of it rapidly becomes computationally infeasible. The number of features was therefore reduced by eliminating nucleic acid base positions that were present in less than 0.1% of sequences. The resulting matrix was given to the Mothur (Schloss et al., 2009) software package as a template and each of the 16S sequences from GenBank that were not in that release of Silva were aligned to the template by Mothur tool using default parameters. The resulting alignment matrix was then trimmed to the V4 region resulting in 320 nucleic acid positions (features) and 117,161 unique V4 sequences (samples).

To create the latent sequence space for V4, the pairwise distances among each of the aligned V4 sequences in the V4 matrix (D) must be accurately reflected in the corresponding distances among those samples in the latent space (d). First, each V4 sequence was converted to a one hot vector, and then the distance to each of the other V4 sequences was calculated based on the alignments and the data was sorted by this distance. The distance (D) reflects the distance between the aligned sequences and its detailed calculation is presented in the Supplementary Material Choosing Proper Distance Metrics. Since it is not computationally feasible to iteratively train the latent space while rigorously adjusting all of the embedded latent space distances (d) for every possible pair during every iteration, a sampling method was used. In order to optimize the latent space to distinguish single base pair variation, the 300 sequences most similar to each V4 sequence based on the distance (D) were included in the sampling. In addition to those 300 nearest sequences to the current V4 sequence, 200 more were chosen for comparison to the current sequence by sampling from the remaining 116,860 potential pairs using a dilation formula that biases toward the most similar remaining sequences with the most distantly related sequences receiving the lowest sampling representation. The intention is to provide sufficient accuracy to distinguish closely related sequences that may differ by as little as a single base using the embedding distance d.

The mathematical details for the sampling method are given here. For each known V4 sequence:

1. Convert the sequence to a one-hot vector, calculate and sort by the distances from this known sequence to all other V4 sequences, selecting the *n* sequences with smallest distance to this known sequence
2. Gather additional sampling from the remaining *m*–*n* V4 sequences using an exponential schedule such that the *i*th sample is at position *n* ∗ ƒ^*i*^ in the sorted samples, where the dilation factor ƒ is found by solving the relation:
$$N=n\times f^{m-n}\;and\;f=e^{\ln\left(\frac{N}{n}\right)/\left(m-n\right)}$$
  Where in this instance:
*N* = 117,161 unique sequences for the V4 region
*m* = 500 total number of samplings per sequence
*n* = 300 number of nearest neighbors included.

After completing these samplings for the distance comparisons that will be used to ensure the constructed latent space reflects the actual sequence distances, the next step was to actually construct the space. Within the latent space, each of the 117,161 V4 sequences was represented as a 10-dimensional vector. Initial values for each vector were filled at random using a centered Gaussian with sigma = 10. Determining the accurate placement for each unique V4 sequence vector in the latent space was done during an iterative gradient descent training by adjusting the distances (*d*) among pairwise sequences within the 10-dimensional latent space to closely match the distances calculated for each of the sampled 500 distance comparisons in the original sequence matrix (*D*). Thus, for each V4 sequence, a total of 500 pairwise comparisons were used in each iteration of the gradient descent training to construct the latent space.

In mathematical terms, given the input sequence space *S* and the embedding space *E*, we seek a mapping f :S→E,  such that for every x,y∈ S and f (x),f(y) ∈  E , we obtain  D (x,y)≈d (f(x),f(y)), where *D* and *d* are distance functions in the original sequence and embedding spaces, respectively. To ensure that *d* corresponds to *D*, we used a loss function that favors nearest neighbors. The form of this loss to be used during gradient descent training is $L={\left(1-\frac{d}{D+\epsilon }\right)}^{2}={\left(\frac{d-D}{D+\epsilon }\right)}^{2}$ . In addition to the accuracy promoted by the sampling approach described above, this loss function will also encourage high resolution for close sequences (small values of *D*) for the facilitated detection of single nucleotide base changes, while permitting lower resolution between highly divergent sequences. The ϵ ∼ 1 regularizes the loss for vanishing phylogenetic distances.

The gradient descent training iteratively continued adjusting distances within the space until the changes to the average loss function with each iteration fell below five significant digits. Finally, we note that the metadata was carried through the process since each Silva/Genbank sequence in the matrix was a known V4 sequence, so each 10-dimensional vector used in the training was associated with a known V4 and its taxonomic identity. Additional mathematical and algorithmic details for the calculation of *D* and for the Gradient Descent and its subfunctions are given in the Supplementary Material.

**FIGURE 2:**
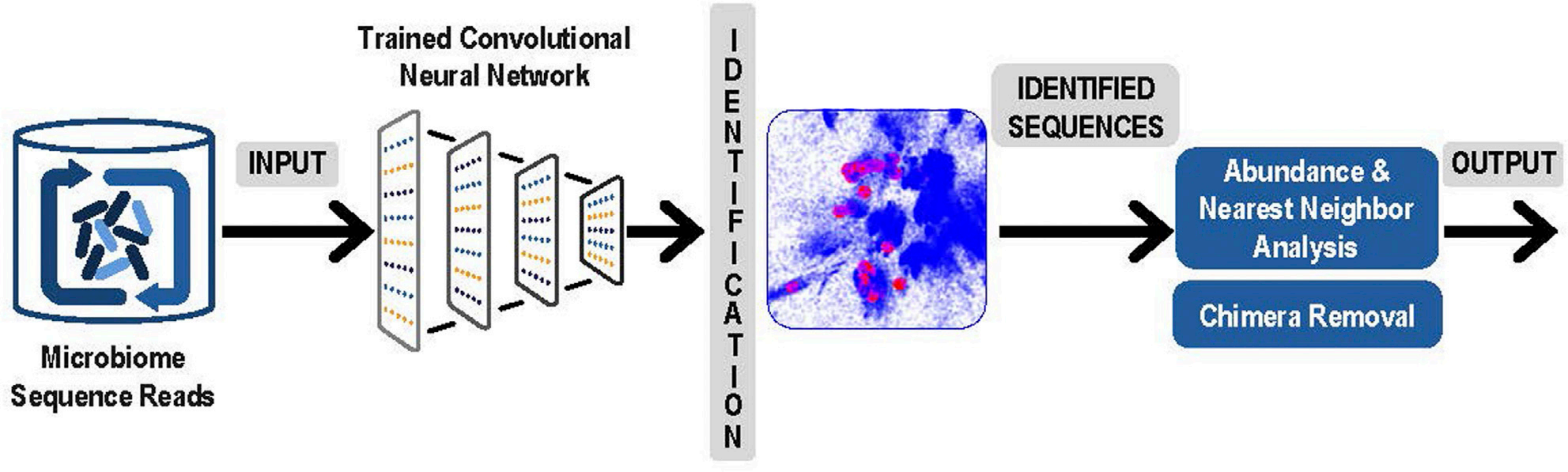
Analysis of Experimental Sequence Data: Paired reads from microbiome data were input to the trained neural network for identification. Resulting clusters were analyzed for abundance and correction of likely sequencing errors resulting in output of each unique ASV and its corresponding abundances.

2. The second step was to train a deep learning algorithm so that it could take any V4 sequence and map it into the previously built latent space. A convolutional neural network was trained using the 117,161 known unique bacterial V4 sequences and their corresponding 10-dimensional vectors in the sequence space. This V4 encoder was a subtype of convolutional neural networks that is fully convolutional (e.g., Long et al., 2015; Maggiori et al., 2016) sometimes also referred to as a fully convolutional network (FCN) with a total of 90 layers, 32 of which were convolutional. A max-pooling layer was inserted after the first two convolutions to reduce the size of the network and encourage translational invariance for spatial motifs. All convolutional layers (except the last) were followed by batch normalization to stabilize training, and a ReLU activation. The final convolution produced the 10-dimensional vector encoding that matched the target 10-dimensional vector for each input sequence. We show a spreadsheet with all 90 layers in the Supplementary Material. As for all trained deep learning algorithms, the trained neural network can generalize and produce 10-dimensional vectors even for sequences not included in the training set, in this case, if new and previously unknown V4 sequences are found experimentally. We trained this CNN encoder within the PyTorch framework using an Adam optimizer with learning rate = 0.0001, a loss function that was simple Euclidean distance between the 10-dimensional output and the precomputed 10-dimensional embeddings. Each training batch of 200 training samples were randomly sampled from the 117,161 V4 SG dataset, training with a total of 50,000 batches.

3. The next step was to use the latent space and the trained convolutional neural network to identify and measure the abundance of the sequences obtained from microbiome experiments. Sequence obtained from paired reads from a microbiome sample were presented to the trained convolutional neural network and mapped into the correct position in the latent space. Note that this now comprised a rapid classification process that was accomplished without any explicit pairwise alignment of the sequences from the microbiome experiment. After each sequence was mapped, the result was a collection of sequences from the microbiome experiment represented as clusters in the latent sequence space. In Figure 2, below we present an overview of this step, and of the next and final step in the process, the denoising.

4. The final step was denoising by examining the experimental microbiome data for possible sequencing errors and finalizing the number of V4 ASVs and their abundance. To separate actual sequence variants from sequencing errors, our denoising process began with analyzing the relative abundances of closely related sequences. Reads with sequence that occurred only once in the microbiome sample were eliminated. For each remaining sequence, a determination was made whether to consider it a valid unique bacterial sequence variant or if it likely originated as a sequencing error from a more abundant “parent” sequence. To identify candidate parent reads, a fast Nearest Neighbor search was done in the latent space using NanoFlann (https://github.com/jlblancoc/nanoflann). A maximum of 20 nearest neighbors were selected that were within at least a 15 bp radius in the latent space and that were at least 20-fold more abundant than the sequence under consideration. For each of these parent candidates the edit distance was computed using Edlib package (Šošic and Šikic, 2017) and only candidates with less than 1 bp difference per 64 bases (98.5% match) were retained. If a sequence had no candidate parents after this process, it became an identified V4 ASV. Otherwise, remaining candidate parents were sorted by edit distance and the closest was selected as the likely parent (in event of a tie, the more abundant was favored). The child sequence was now considered to be a likely sequencing error originating from the parent sequence. Once the process was completed, the parent-child relationships were traversed until a sequence was identified that had no parent. Each sequence that had no parent was then considered an identified V4 ASV and its children and any grandchildren were then included into its abundance count.

Standard chimera removal was accomplished using the VSEARCH package uchime_denovo command. Since the latent space training in the previous step had been conducted with known V4 sequences, and the phylogenetic metadata from the Silva/Genbank sequence matrix was carried through for each 10-dimensional vector during that latent space training, the 10-dimensional vector for each resulting ASV in the latent space was readily associated with phylogenetic information for its exact or closest-associated known V4.

### Analysis on Mock Community Data Sets

Two sets of experimental microbiome data for mock communities were then analyzed using these methods. First, the data were processed as in step 3 above and mapped by the trained convolutional neural network. Next, they were processed through step 4 for denoising and finalization of the ASVs. These data sets were chosen from the mockrobiota resource (Bokulich et al., 2016; http://caporaso-lab.github.io/mockrobiota/) for samples that were created by sequencing bacterial mixtures of known abundance and composition in order to rigorously assess the performance of the method by using data for which the results to be obtained are known. Mock 16 (Schirmer et al., 2015) was selected to determine the robustness of the method for identifying ASVs on a complex mixture of known composition, containing 49 bacterial and 10 archaeal species. Mock 12 (Callahan et al., 2016) was chosen to examine the method across extreme variation in concentration across the bacterial mixture. The Mock 23 (Gohl et al., 2016) was chosen to give additional statistical basis for abundance estimation and average speed measurements on a relatively small and less complex data set. Since it does not exceed the Mock 16 for diversity or the Mock 12 for concentration range, the analysis in addition to speed is shown in the Supplementary Tables S5, S6. Each ASV identified for each data set was validated by BLAST to confirm DERSI’s taxonomic identification.

In most cases, the bacterial genomes contain more than one copy of the 16S gene, and these additional copies may be identical or varied in their V4 region sequences. In order to provide a high level of rigor to the expected sequences and their abundances, we therefore deemed it necessary in our analysis to calculate the expected number of V4 sequences by looking at the genomes of each bacterium and to adjust the expected number of ASVs for each bacterium. In some cases, the V4 sequences were identical between two different bacterial genomes, and the number of expected ASVs was accordingly reduced. In a few cases, full bacterial genomes were not yet available and best estimates were made based on numbers and sequences of closely related bacterial genomes. The expected numbers of ASVs and OTUs depends upon knowing how many variants are present in each genome. And while copy number does not affect the number of ASVs, it does affect the expected abundance measures. Therefore, both the number of expected ASVs and their expected % of the total composition were adjusted to reflect the genomic composition of the mock community.

While these mock community data sets are intentional compositions, previous work on these data sets has also demonstrated the presence of unintentional contaminants (e.g., Callahan et al., 2016; Edgar, 2016; Nearing et al., 2018). Calculating precision and recall for this type of data requires determining exactly how a useful number can be rigorously generated, given that unintended contaminants are present in these data. A prior review of microbiome algorithms chose to calculate precision based on perfect matches to a reference sequence considered true positives versus noisy (less than 100%) matches to known sequence considered false positives (Caruso et al., 2019), and we have based our approach largely upon this. A second method was also presented that considered all unexpected sequences to be false positives, and therefore was likely to confound the accuracy of the experimental protocol and its susceptibility to contamination with the accuracy of the algorithms.

At very low concentrations it becomes very challenging to assess false positives versus minor contaminants, and VSEARCH has been previously shown to identify large numbers of such sequences. Given the difficulty in assessing whether this shows exceptional sensitivity to low abundance contaminants or a severely elevated false positive rate, we chose to apply several filters to the sequences that do not have a 100% match to a known V4. We eliminated ASV/OTUs: 1) with less than 92% identity to the closest known V4 sequence; 2) or with less than 0.01% abundance and less than 99% identity to the closest known sequence; 3) or with less than 0.001% abundance and less than 100% identity. At this level of stringency, those V4 sequences found at very low concentrations are highly likely to be false positives since at 99% identity they will be only one or two bases different from a known V4 sequence.

Of the remaining ASVs/OTUs those identified at 100% to a known sequence are considered to be true positives whether intentionally added to the mock community or not. The complete set of sequences to be used for calculating recall numbers was the union of all such unique V4s with a 100% match to a known sequence found by all four compared algorithms.

Since we have conducted a genomic analysis to identify expected values for each of the V4 sequences from the intentionally input bacterial genomes, we are able to make a comparison of the identified values to the expected values. A Bhattacharyya coefficient (Bhattacharyya, 1943) was computed over the abundances detected for both mockrobiota data sets to give a measure of the overall accuracies of the abundance estimates made by each algorithm. The Bhattacharyya coefficient provides a divergence measure between two multinomial populations and so is suitable to describe the differences between the population of expected sequence abundance values for the input mock community with the second multinomial population being the values of those sequence abundance values reported by the algorithm being tested.

### Data Sets for Speed Comparison

The Mock 23 was included to give additional statistical basis for average speed measurements on a relatively small and less complex data set. We also included the Mock 12 and Mock 16 data, and these show increasing size.

Finally, to assess the speed performance on much larger data sets than afforded by these mock communities, we selected the first 1,072 samples from the Goodrich et al. (2016) microbiome data set. Since this is a human biologically identified data set the exact expected composition of the bacterial community and associated abundances are not known and therefore a full analysis against expected values was not performed, it was only used for the speed comparisons.

### Data Sets for Phenotypic Analysis

The motivation for the development of the DERSI method was greater accuracy in the determination of ASVs combined with high speed. However, to demonstrate that this approach also supports phenotypic analysis, we chose several previously published data sets for which to compare to published results for experimental data sets where the exact composition of the bacterial mixture is not known but the correct phenotype for the overall sample is known.

We selected two microbiome datasets that used 16S V4 sequence from an analysis of the effects of water decontamination method and choice of bedding material on the fecal microbiome of mice (Bidot et al., 2018). We selected the first data set of fecal microbiome samples for mice in which both groups use corncob bedding but one group was given autoclaved water and the other group water purified by reverse osmosis. The second data set of fecal microbiome data was from mice who were given either paper bedding and water purified by reverse osmosis or corncob bedding and water purified by autoclaving.

The third set of microbiome data was chosen from Mezzasalma et al. (2018) comprising multiple microbiome samples from three grape cultivars grown in the same vineyard. Each sample was taken from a different vine and consisted of consisted of a small bunch of grapes. The cultivar was the phenotype to be predicted. The original V3–V4 reads were trimmed to obtain the V4 sequencing data from this experiment.

A fourth set of microbiome data for phenotypic analysis was taken from a study of chicken ceca transplantation (Glendinning et al., 2022). Microbiome samples from two ceca obtained from donor chickens of the Roslin broiler breed were transplanted into chickens of the Ross broiler breed. Additional chickens received sham transplantations with saline as controls. Subsequently, the microbiome samples from the transplant recipient chickens, the two donor ceca and the controls were sampled and sequenced using the V4 region of the 16S.

For all phenotype data sets, the V4 sequence reads were input to the DERSI trained convolutional neural network. The output data was then normalized using a trimmed mean and taking the logarithm, followed by the denoising process described in step 4 above. PCA was then used to map each normalized sample into a 3D space for comparison to published results.

### Benchmarks

The analysis of the diverse Mock 16 and the extreme concentration variation Mock 12 data sets was benchmarked against three widely used methods for microbiome analysis: an OTU method, VSEARCH (Rognes et al., 2016) and two ASV method DADA2 (Callahan et al., 2016) and UNOISE3 (Edgar, 2016). VSEARCH was chosen since it has been widely used for OTU analysis, has been benchmarked against other algorithms and is included in the QIIME2 microbiome pipeline. DADA2 has been widely recognized as the most sensitive method for detection of ASVs in multiple benchmarks and is also included in the QIIME2 pipeline where it can be optionally selected. UNOISE3 is private source software with a freeware 32-bit executable and has been shown to give near comparable results to DADA2, sometimes with greater specificity. VSEARCH and DADA2 were run using QIIME2, and the 32-bit UNOISE3 software for Linux was downloaded from https://drive5.com/usearch/download.html as part of the overall USEARCH package.

Speed for all four methods was measured on the same System76 Oryx Pro Laptop using a Linux operating system (Ubuntu 20.04). Multiple steps were included in the speed measurements, including preprocessing and dereplication, identification of ASVs/OTUs, denoising to correct potential sequence errors, chimera removal and abundance calculations.

### Parameters for Each Algorithm for Analysis and Speed Comparisons

These are the details of the steps and parameters used for comparison of the algorithms for the mock community analysis and the speed comparisons. Primers were removed from the mock community data sets Mock 16 and Mock 23 using multiple sequence alignment against our expanded Silva database using mothur. Primers were not present in Mock 12. Pooling of samples is an option for some algorithms, however, we did not pool samples for these benchmarks in order to be close to DADA2’s process and ensure a fair comparison.

DADA2 was run as included with its particular preprocessing methods and defaults in QIIME2 except for forward and reverse quality trimming. The only parameters changed were the forward and reverse truncation, determined by inspecting Q values for each data set (all other parameters were left at their defaults):

mock12 -p-trunc-len-f 180 -p-trunc-len-r 140
mock16 and mock 23 -p-trunc-len-f 200 -p-trunc-len-r 180
Goodrich study -p-trunc-len-f 200 -p-trunc-len-r 140.

For VSEARCH, UNOISE3 and DERSI, after removing primers, we performed the identical merging and quality filtering so they would each receive the same input. This was accomplished using the vsearch command --fastq_mergepairs to merge pairs, with the following settings: -fastq_ascii 33; --fastq_minlen 180; --fastq_minovlen 20; --fastq_maxdiffs 12; --fastq_qmin 0; --fastq_qminout 0'; --fastq_qmax 41; --fastq_qmaxout 41; --fasta_width 0; --fastq_maxns 0. We then used the vsearch command --fastq_filter to quality filter, with following settings: --fastq_maxee 1.0; --fastq_minlen 225; --fastq_maxlen 275; --fastq_maxns 0; --fasta_width 0.

The above two steps were common to VSEARCH, UNOISE3 and DERSI to ensure that each received the identical input to dereplication. Each algorithm used its own dereplication method but VSEARCH, USEARCH (UNOISE3) and DERSI’s dereplications are equivalent. All three algorithms dropped singletons. Reads were dereplicated using the vsearch/usearch command: --fastx_uniques --minuniquesize 2. OTUs/ASVs abundances were produced for VSEARCH with the following commands: vsearch --cluster_size {} --id 0.97 and then vsearch --uchime_denovo. For UNOISE3 the USEARCH commands: usearch -unoise3 (all default settings; this does error correction) usearch -otutab (this constructs abundances querying original sequences including singetons). For DERSI, dereplicated reads were encoded by the neural network, and these embeddings were then used for error correction as described in step 4 above. Chimera removal used vearch --uchime_denovo.

## Results

### Latent Space Creation and Use

Training during Step 1 of the method continued until the loss function achieved an average value of 0.1101 and the variation at each iteration fell below five significant digits. The resultant latent space is a dense, structured, 10-dimensional point cloud for all known V4 sequences that reflects their aligned distances from each other to a very high degree of accuracy.

**FIGURE 3:**
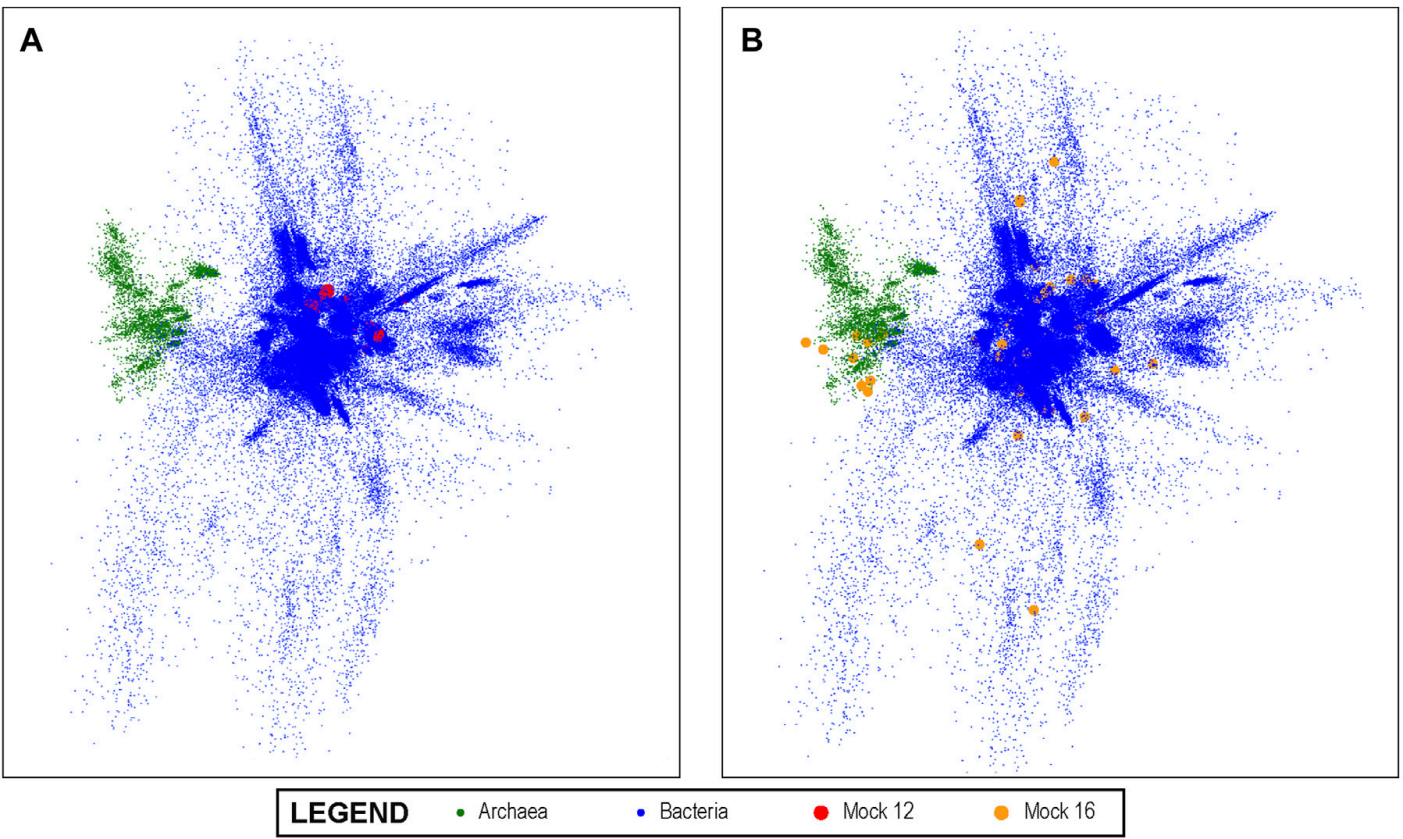
The 10-dimensional V4 Latent Space: The latent space of all known bacterial V4 sequences projected into three dimensions using PCA for visualization; the Archeal V4s clearly separate from other V4s. In **(A)** we show the projection of the mock 12 community sequences as mapped by our V4 encoders onto the overall bacterial latent space. It can be visually observed that the mock 12consists of a relatively small number and not especially diverse set of bacteria. In **(B)**, we projected the highly diverse mock 16 community as mapped by the V4 encoder and this much greater diversity is readily observed. This demonstrates the ability of the method to produce results that can offer informative visualizations.

A visualization of this space is shown in Figure 3 using PCA to project the 10-dimensional space into three dimensions. In the visualization, each dot represents a unique V4 sequence, and its proximity to other dots accurately reflects their sequence similarity. It can be seen that there are distinguishable groups of closely related sequences. Archaea, for example, are shown in green and are clearly more closely related to each other than to other V4 sequences, as would be expected from molecular phylogeny. Since the latent space created by this method lends itself to this type of visual representation, it also enables the results of the analysis of experimental data sets that are mapped into this space by the trained convolutional neural network to be projected onto this overall visualization. We show this in Figure 3A for the Mock 12 data set and in Figure 3B for the Mock 16 data set. It can be visually observed that the Mock 16 represents a highly diverse set with members distributed widely over known bacterial genome space. In contrast, the Mock 12 data set that consisted primarily of Bacterioidies and Firmicutes, shows a much more compact distribution.

### Analysis of the Mock-16 Data Set

For the initial evaluation and testing of our new V4 deep learning encoding approach DERSI, as well as for comparisons to the other widely used identification and quantitation algorithms DADA2 UNOISE3 and VSEARCH, we chose the Mock-16 data set because of its significant phylogenetic breadth, as it contains 59 species, 10 of which are Archaea. Although DNA from each of the 59 species was added in equal amounts, bacterial species vary in the copy numbers of the 16S gene and may have sequence variation among those copies for the V4 regions. Some species also share identical V4 regions. We have therefore calculated the expected number of unique V4s in the mock community as 63, details are shown in Supplementary Material.

A comparison of the output from analyzing the Mock-16 data set with DERSI, DADA2, UNOISE3 and VSEARCH is shown in Table 1. As detailed in the *Materials and Methods* for the calculation of precision and recall, some sequences identified at very low concentrations and with no exact matches to a known sequence were eliminated from consideration since it cannot be determined if they are false positives or represent actual contaminants of novel sequences in very low amounts.

**TABLE 1:** Algorithm performance on complex bacterial mixture in the mock 16 data.

V4 variants	DERSI	DADA2	UNOISE3	VSEARCH
Added to Mock 16 Found/expected	60/63	59/63	60/63	48/63
Contaminants (>0.001%) Found/expected	21/22	21/22	19/22	17/22
False Positives (>0.01%) Found	1	6	1	3
Precision/Recall	99/95	93/94	99/93	96/76

Each ASV identified was validated by BLAST to confirm DERSI’s taxonomic identification, and all taxonomy was found to be accurate. In our Supplementary Material, we provide the details of how each unique ASV identified maps to a bacterial species or group.

The output from DERSI, UNOISE3 and DADA2 shows virtually the same ASVs until the read count is below 31 reads per ASV. This is ∼0.006% of a very large read count for a single sample (∼520,000 reads). Below this level each algorithm finds a slightly different set of ASVs. DERSI finds 14, DADA2 finds 12, VSEARCH finds 11 and UNOISE3 finds 12, with DERSI retaining a slightly higher fidelity at these lower levels. We also note that in some cases with this set of experimental data the sequencing method itself failed due to no/low productivity of certain primer sets resulting in lack of detection by all three algorithms. It has been previously noted that the primers used for the V4 region do not amplify all V4s with equal efficiency resulting in some ASVs or OTUs that were not found in the sequence data (Allaband et al., 2019).

VSEARCH tends to combine very closely related ASVs into a single OTU, which can obscure the presence of closely related species, for example, it has put *Chlorobium phaeobacteroides strain DSM 266, Chlorobium phaeovibrioides DSM 265 and Chlorobium limicola strain DSM 245* into a single OTU (Supplementary Material). This is in keeping with the VSEARCH algorithm intended to predict at an OTU level rather than at the finer-grained prediction of ASV methods.

Row 2 of the table contains ASVs that were not intentionally included in the mock community but are detected and have 100% match to a known bacterial sequence. These are not closely related to the original input organisms and should not be considered false positives, but likely arise from inadvertent contamination. Many of these have been previously described (Callahan et al., 2016) in the original analysis of the data set. We have therefore included them in the precision and recall analysis. We note that our algorithm DERSI does slightly better at detecting such potential contamination.

Overall, VSEARCH performs considerably less well than DERSI. While UNOISE3 and DADA2 perform relatively well, DERSI has the best precision and recall of the four algorithms on the mock 16 data set.

### Analysis of the Mock-12 Data Set

For the second major test of our new algorithm, we analyzed the data set listed as Mockrobiota Mock-12 since it has a 5-log unit variation in the input abundances (see Table 2 first column). As was true for Mock 16, the exact expected abundance may vary from the input percentage of the bacteria due to multiple copies within a genome and sequence variation in these multiple copies (see Supplementary Table S3 for details). Three of the five genomes in the most abundant two categories and two of the genomes in the lowest abundance category have multiple V4 regions. A number of the species input still do not have a complete genome in GenBank and in those cases the copy number was estimated based on closely related genomes. We show the resulting likely number of input V4s in Table 2 as the expected count. The read abundance data does not vary significantly from the expected abundance based on this approximation (for a full list of expected abundances for each bacterial species, see the Supplementary Material). Each ASV identified was validated by BLAST to confirm DERSI’s taxonomic identification, and the taxonomy provided was found to be correct.

**TABLE 2:** Algorithm performance across extreme abundance variation in the mock 12 data.

V4 variants	DERSI found/expected	DADA2 found/expected	UNOISE3 found/expected	VSEARCH found/expected
Added at >10%	2/2	2/2	2/2	2/2
Added at 1–10%	7/7	7/7	7/7	3/7
Added at 0.1–1%	4/4	4/4	4/4	4/4
Added at 0.01–0.1%	4/4	3/4	4/4	2/4
Added at 0.001–0.01%	4/4	3/4	4/4	3/4
Added at 0.0001–0.001%	7/13	4/13	0/13	7/13
Contaminant at 0.001–0.01%	2/2	2/2	2/2	2/2
Contaminant at 0.0001–0.001%	10/10	2/10	0/10	8/10
False positive at 0.01–0.1%	0	7	1	1
False positive at 0.001–0.01%	0	1	1	1
Precision/recall	100/87	77/67.5	92/50	94/67

The analysis of the four algorithms follows similar outcomes to that seen in the Mock-16 analysis. DERSI and UNOISE3 create an identical list of ASVs until the read count is below 17 or 0.0012%, at which point DERSI performs significantly better. Whereas VSEARCH tends to combine closely related V4s into single OTUs (more details in Supplementary Material). DERSI and UNOISE3 find two genomes, one (*Bacteriodes fragilis*) at the 0.01–0.1% abundance category and one genome (*Eubacterium rectale DSM 17629*) in the second lowest abundance category that DADA2 folded into another ASV. UNOISE3 misses all, while DADA2 misses four of the genomes in the lowest abundance category. DERSI and VSEARCH find seven of the 13 of the lowest abundance ASVs or OTUs in the input data set; note that none of the algorithms find any of the other six, they appear to be missing from the set of reads.

In rows 7 and 8 of Table 2, we present a summary of sequences identified in the mock community that were not expected based on the intended bacterial inputs but match a known V4 at 100% identity, and therefore likely represent contaminants. These appear only at the two lowest concentrations. All four algorithms find the two most abundant contaminants (*Enterococcus hirae* and *Anaerostipes caccae*). Whereas, DERSI finds 10, VSEARCH 8, DADA2 two and UNOISE3 0 of the low level contaminants.

As shown in rows 9 and 10 of Table 2, DERSI identifies 0, UNOISE3 2, VSEARCH 2, and DADA2 eight ASVs or OTUs that have no known match at 100% and meet the criteria for false positives.

The calculated precision and recall show that DERSI has the highest precision and the highest recall of the four algorithms. In fact, DERSI achieves 100% precision results on the Mock 12 for ASV identification, and for recall, DERSI misses only six of the lowest abundance V4s that appear to be actually missing from the input sequence data.

### Abundance Analysis

In Table 3, we present a summary of the accuracy of all four algorithms in correctly identifying the known abundance across the full mock 12, 16 and mock 23 data sets. Expected values were created by examining both copy numbers of the 16S gene, and whether the bacterial genomes contained multiple copies, some of which may be variant within the V4 region.

**TABLE 3:** Bhattacharyya coefficient comparing abundance estimates to expected values.

Data set	DERSI	DADA2	UNOISE3	VSEARCH
mock 12	99.78	99.61	99.79	87.77
mock 16	96.24	96.00	96.29	91.12
mock 23	98.86	98.45	98.75	98.75

To rigorously compare the results for each algorithm to the expect values we applied the Bhattacharyya coefficient. The Bhattacharyya coefficients computed for each algorithm compare the abundances to the expected values for the input mix of bacteria over the entire data set; higher scores are better. Details of abundances for each individual bacterium in each data set can be found in Supplementary Material. VSEARCH does not perform as well as the other algorithms, likely due to the OTU approach to grouping. The accurate performance of DERSI is nearly identical to UNOISE3 exceeding UNOISE3 by only 0.02 overall, an amount that is statistically insignificant. DERSI does very slightly better compared to DADA2 in reproducing expected abundances, but there are only very slight variations among the three algorithms.

### Benchmark for Speed

We compared our algorithm, DERSI, to DADA2, UNOISE3 and VSEARCH on four data sets using the same laptop and present the results in Table 4. The size of each data set is given by the number of V4 sequence reads. One of the known advantages of the OTU algorithm VSEARCH is its speed, and indeed VSEARCH shows the best performance across all data sets. Of the ASV methods, our algorithm DERSI was the most rapid. We note that UNOISE3 is initially faster than DADA2 but loses this advantage for the largest data set. The UNOISE3 denoising algorithm itself is a very rapid step but outputs only a list of ASVs without abundances. The step to determine abundances in the USEARCH package is much slower than the error correction but is included in the measure since DERSI outputs both a list of ASVs and their abundances as do DADA2 and VSEARCH. We conclude that DERSI offers a speed advantage across a broad range of data set sizes.

**TABLE 4:** Speed in Seconds of Each Algorithm on four data sets.

Data set	Sequence reads	DERSI	DADA2	UNOISE3	VSEARCH
mock23	329,358	13	316	15	5
mock16	592,231	22	427	60	11
mock12	2,040,485	48	813	93	29
Goodrich	467,643,460	7,548	12,387	21,080	847

### Phenotypic Prediction

Since DERSI was designed and optimized for accuracy in identifying ASVs and their abundance, it is desirable to show that this approach is still able to support phenotypic prediction that relies on all the data for each sample as a whole. There are no phenotypes associated with mock communities so to examine the effectiveness of DERSI on experimental data of unknown bacterial composition, but known sample phenotype, we reanalyzed four published data sets to compare phenotypic predictions to published results. All results are shown as scatter plots using the first three principal components produced by PCA.

**FIGURE 4:**
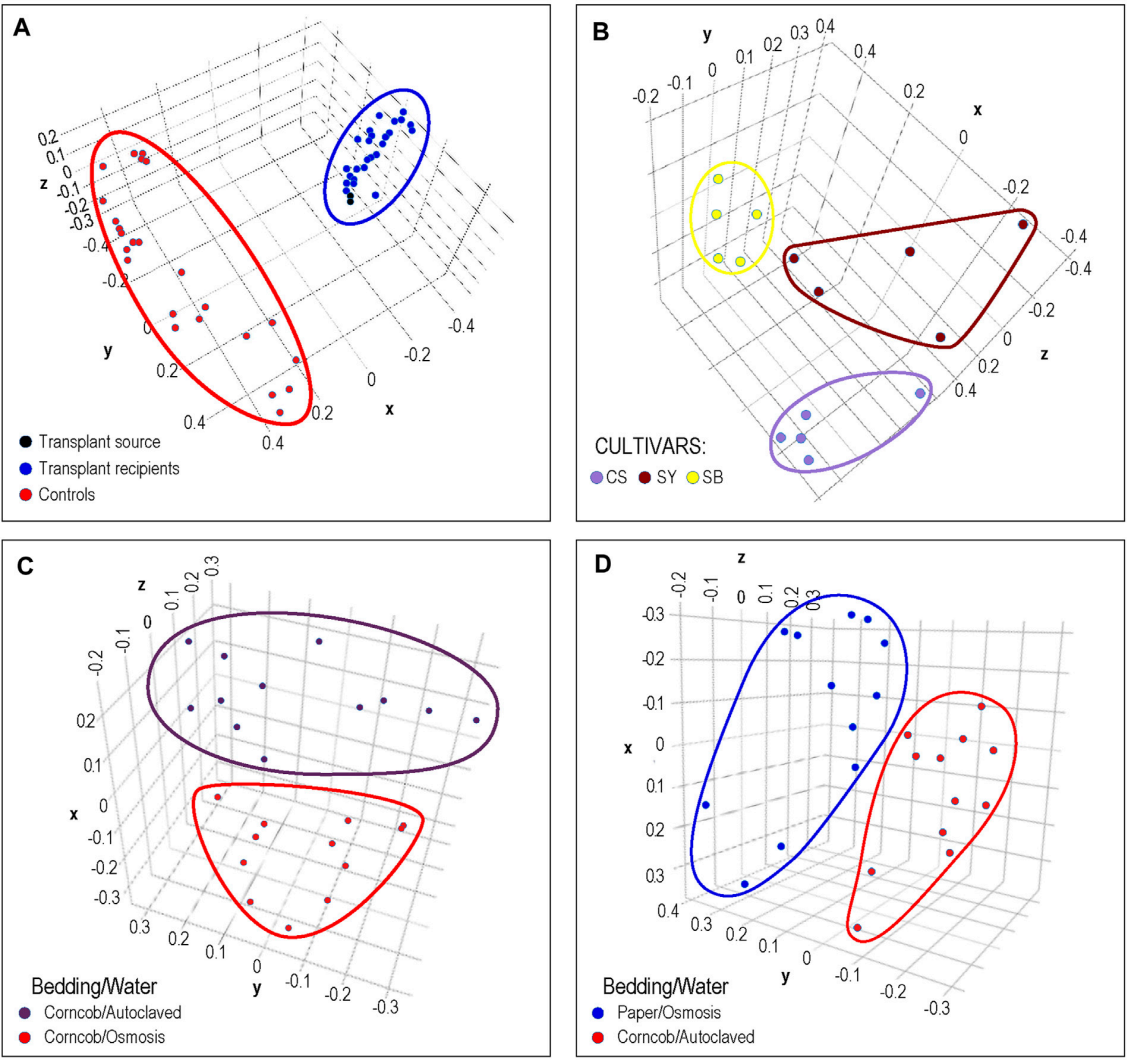
Phenotypic Analysis: Each dot represents a sample from previously published microbiome data that was processed by DERSI into a set of ASVs. These were then normalized and plotted using PCA. The axes represent the first three principal components. In **(A)**, we the results of a chicken cecal microbiome transplantation experiment. Clear separation was achieved between controls and the transplant recipients whose microbiomes cluster with their donors. In **(B)**, we show a grape microbiome experiment; each cultivar sample was a small bunch of grapes collected from the Alpine Italian Vineyard. Each cultivar is linearly separable, exceeding the results in the original publication in which two of the three cultivars overlapped. In **(C)**, we show the impact of choice of water purification method on mouse microbiomes, the two groups of microbiomes are separable. In **(D)** we show the separation of microbiomes of mice using paper bedding with osmosis purified water in blue, to microbiomes of mice using corncob bedding and autoclaved water. Although the separation is relatively narrow, the distance between groups does exceed that of the original publication. In all of these cases, DERSI’s output matched or exceeded that of previously published results showing that the method does support phenotypic analysis.

In Figure 4A, the results of cecal transplants between breeds of chickens are presented. One group (shown in red) represents sham transplants using saline solution and are the same breed as the transplant recipients. The other group shows two cecal transplant donor microbiomes in black from a different chicken breed than sham and actual donors, and the transplant recipients in blue. It can clearly be seen that the microbiome of the recipients is very similar to the donor microbiomes with which they group. This matches the results shown in the original publication.

In Figure 4B, we show the normalized results from DERSI on the grape cultivar microbiome data (Mezzasalma et al., 2018). The microbiomes from the three cultivars are linearly separable. Our analysis shows that our increased accuracy for sequence variants and abundance clearly also supports effective phenotype prediction since it readily separates the microbiomes for all samples from the three different cultivars in the Italian Alpine Vineyard, whereas in the initial published analysis only one cultivar (Sauvignon Blanc, yellow) could be cleanly separated from the other two.

In Figures 4C,D, we show the results of phenotypic prediction on two microbiome datasets from an analysis of the effects of water decontamination method and choice of bedding material on mice (Bidot et al., 2018). In C, a PCA of the microbiome composition for two phenotypic groups, mice who shared the same bedding type but whose water was purified either by osmosis or by autoclaving. The two water phenotypes clearly assort from each other and are represented by two distinct groups. This meets or slightly exceeds the separation shown in the original publication. Similarly, in the panel at right we show mice who used paper bedding with osmosis purified water compared to the microbiomes of mice who used corncob bedding and autoclaved water. The separation between the two groups appears to surpass that in Bidot et al. (2018).

Taken together, all four results support the conclusion that the accuracy for sequence variants and abundance shown for DERSI also supports quality phenotypic analysis that can match or exceed published results.

## Discussion

To our knowledge, this is the first demonstration of the use of latent space with a deep learning algorithm to make sequence identifications, and moreover to be able to distinguish closely-related sequence variations with single base accuracy. The first step is a gradient descent training to form the latent space. This 10-dimensional space is an embedding of all of the sequences that reflects their phylogenetic distance and is far more information-rich than a typical two-dimensional phylogenetic tree while still having reduced dimensionality. It becomes a reference component of the method and is not repeated when microbiome samples are being analyzed. This training need only be redone when the collection of known V4 sequences has grown to the extent that the reference space needs to be refreshed.

The process of training the convolutional neural network similarly creates a tool that becomes a stable part of the method and is not repeated with each analysis run. Any machine learning effort can be divided into two phases: 1) a design and construction phase using training data and 2) a deployment phase for predicting on new data. The first phase is where the permanent structure and elements of the tool are decided. For DADA2, VSEARCH, and UNOISE3, the equivalent process consists of algorithmic structure but without any pre-trained results; they must establish transitions probabilities (DADA2) or k-mer features (VSEARCH, UNOISE3) with every analysis run. In the example of a neural network, including our convolutional neural network, an architecture is chosen by a human before any processing of data and then the network weights are fixed by the training procedure. This construction phase is done only once for DERSI. Thereafter the NN runs in its deployment phase, and it is standard procedure to assess neural network speed and performance including only its inference on new data, as we have done on the four data sets for the benchmark. While there is additional time devoted to the original development of the tool, for the runs on data, DERSI was clearly the most rapid algorithm.

Some run time choices may also affect speed. We provide end to end processing time for speed tests, from fastq files to OTU/ASV abundance output, since this is the time a user will experience running these algorithms. To be consistent with DADA2, we employed denoising on a sample-by-sample basis for VSEARCH, USEARCH UNOISE3, and DERSI (rather than the much faster pooling of all reads into a single “super sample”). The sample-by-sample approach helps preserve ASVs that might otherwise be folded into close and more abundant variants. On the other hand, besides speed, the pooling approach does have the benefit of suppressing false positives (along with some true positives just mentioned), on average elevating signal over noise. Some advantages and disadvantage of pooling of samples and sample-by-sample analysis are further discussed by Edgar, (2016). The choice can largely be experimentally driven. Both DERSI and UNOISE3/USEARCH could utilize the pooling of samples instead of the single sample approach we have used for the benchmarks here, and that would likely greatly enhance the speed of both relative to the other algorithms.

We also note that our DERSI process is single threaded. The other three algorithms are implemented in their software as multi-threaded, so that much of their process can run in parallel. There is no algorithmic barrier to multi-threading the DERSI algorithm and that would also further enhance its speed.

Programming language choice and operating system may also impact speed. Marrizoni et al. (2020) compared several microbiome pipelines on two different operating systems and found some differences in actual results among versions of the same pipeline available for Mac OS and Linux. Deep learning algorithms are also able to readily leverage GPUs which are fast relative to CPUs, but it is unlikely that the others used in our comparison could do so to great advantage.

Our convolutional network maps each sequence to the 10-dimensional space that has been previously optimized to capture both global and local phylogenetic sequence structure associated with a large rRNA V4 database. Even without further potential enhancements, it is this approach that enables the analysis of each V4 data set to be accomplished with excellent speed, while still providing the best available accuracy.

We are also unaware of any deep learning algorithms being integrated into methods for sequencing error correction, particularly removing sequencing noise while resolving true genomic variations. While our error correction method bears some similarity to the algorithmic approach of UNOISE3 the major difference lies in the fact that clustering to find nearest neighbors, and to seed potential ASVs occurs in the 10-dimensional latent space leveraging the locations in that space that have been assigned to each sequence by the trained CNN. The analogous step in UNOISE3 (and in VSEARCH) uses kmers to find nearest neighbors.

At the time that many mock communities were added to the Mockrobiota resource, not all bacterial species used had full genomic sequence, and a few of the bacteria used still lack complete genomic sequence. Since most bacteria have multiple copies of the 16S gene, we adjusted our expected abundances using not merely the percent of the bacteria that was used in creating the mock community, but also how the genomic copy number and the number of variants affect the expected abundance. In the few cases where the genomes are still not fully known, we used closest relatives to approximate the genome copies. While previous studies compared the abundances found among different algorithms, we did not find any prior work that utilized the genomic information about copy number and number of variants for the genomes intentionally added to the mock community. We also note that we did not attempt to correct the expected abundance percentages given that each mock community appeared to have some bacterial contaminants. There is no accurate way to know the true abundance of the contaminant. Our use of the Bhattacharyya coefficient to compare expected abundances of the intentionally added sequences to the abundances found for them by each algorithm would be expected to slightly lower the scores of all algorithms due to contaminants but would have impacted all of them equivalently so the comparisons would be expected to be valid. Since most of the algorithms performed quite well at abundance estimation, the impact of this appears to be quite small. Moreover, despite the differences among the algorithms in precision and recall, the three ASV methods are all near equally good at abundance estimation, likely because most of the differences occurred at the lowest concentration levels that would least impact the coefficient over the entire data set. While the coefficient showed a slim and likely statistically insignificant advantage for DERSI over the others, the results do clearly demonstrate that DERSI is able to at least match the best available algorithms for accurate abundance estimation.

The dimensionality of the latent space was chosen empirically by starting with three and increasing. We found 10 dimensions achieved high precision and recall, good abundance recovery and equaled or matched the best available current methods for the analysis of the microbiome mock communities. In the future if applying the method to longer sequences, it might become necessary to use a higher dimensionality for the latent space, potentially incurring somewhat higher computational overhead in the one-time training for the embedding of all know sequences of the chosen length, but should not have much impact on the mapping into the space that is a rapid step using the trained CNN that occurs when using the method on microbiome data sets.

Since Zhao et al. intended to improve phenotypic prediction without an intermediate prediction of ASVs, they leveraged deep learning to classify each individual sequence read for its likelihood to belong to a phenotypic class. Our approach was fundamentally different, although our encoder has a convolutional architecture, it is usedfor mapping sequence reads into a latent space that has reduced dimensions. The reduced dimensions of the latent space enables computational efficiencies. Our output is analogous to the separately trained word embeddings that have been a critical ingredient supporting recent advances in natural language processing (NLP). These word embeddings serve as compact representations of word usage that encode the contents of a document while reducing dimensionality and are the input for larger neural network such as BERT (Devlin et al., 2019). Our sequence embeddings are analogous in that they are trained to faithfully represent a biological sequence (instead of a word or phrase). This paper focuses primary on the quality of those embeddings, as judged by their usefulness in recovering true biological sequences in mock communities. In the future, for phenotype studies, it would be possible to develop even more powerful neural networks that leverage these embeddings further for phenotypic classification, just as BERT leverages its word embeddings to classify documents. In the current work, we have demonstrated that a simple purely linear network (i.e., Principle Component Analysis) on the output ASVs for each sample is sufficient to recover the phenotypic structure of the samples and obtains at least equivalent or slightly improved results compared to previously published work.

Moreover, creating a latent space using these methods for the full 16S sequence should enable a 16S latent space to be used with multiple convolutional neural networks, each trained to map a different variable region of the 16S gene into the full 16S latent space. This would offer a significant advancement in the ability to directly compare microbiome studies conducted by sequencing different variable regions of the 16S gene and enabling more informative meta-analysis of the underlying biology, although some caution would be warranted due to technical differences in the amplification of diverse sequences (e.g., Bukin et al., 2019; Darwish et al., 2021). As full-length 16S sequencing becomes more economical and accurate, a convolutional neural network could also be trained on the full length rather than just the variable regions.

In fact, the method should be generalizable to many types of experiments that rely on sequence identification in addition to microbiome analysis, making this a promising area for future research to fully explore the applicability of these methods to additional biological studies. For example, rather than a latent space intended for microbiome analysis, one could be created from all known sequences for any particular protein or enzyme family and applied to proteomics data. In addition, metagenomic analysis is currently very computationally burdensome and accuracy is challenging for low abundance organisms. Potentially DERSI could shift that burden away from the individual metagenomic experiments and onto the one-time creation of a very large latent space and the training of multiple deep learning algorithms. For these more demanding analyses, reduction even in the one-time computational demands of initially creating the latent space could be managed by judicious choice of the metagenomic challenge to address. For example, rather than full genomes, the system could be applied to the phylogenetic classification of metagenomic samples by training a number of individual neural nets on each of a subset of the 92 core bacterial genes identified by the UBCG pipeline (Na et al., 2018).

In conclusion, the current work demonstrates that our Deep Learning for Rapid Sequence Identification (DERSI) algorithm that combines a latent sequence space with a deep learning encoder can match or better the precision and recall of existing widely accepted methods for microbiome analysis while performing at greater speed. Potential exists for further enhancing the speed of the algorithm, and of generalizing the method to more types of data including metagenomics and proteomics.

## Data Availability

Publicly available datasets were analyzed in this study. This data can be found here: The Mock 12, Mock 16 and Mock 23 datasets can be found at http://caporaso-lab.github.io/mockrobiota/. The grape microbiome data sets can be found in the EBI metagenomics portal (https://www.ebi.ac.uk/metagenomics/) under the accession code PRJEB25720 (ERP107664). The remaining microbiome data sets are also from EBI and can be found at that following links: Chicken: PRJEB46338 https://www.ebi.ac.uk/ena/browser/view/PRJEB46338?show=reads Mouse: PRJNA453789 https://www.ebi.ac.uk/ena/browser/view/PRJNA453789?show=reads Twins: PRJEB13747 https://www.ebi.ac.uk/ena/browser/view/PRJEB13747?show=reads.
