# Supplementary material for: Deep Learning Encoding for Rapid Sequence Identification on Microbiome Data
Source: Front Bioinform. 2022 Jun 24;2:871256. doi: 10.3389/fbinf.2022.871256 (PMC9580936; doi:10.3389/fbinf.2022.871256)
Supplement: Supplementary file 2 [file DataSheet4.PDF]

GRADIENT DESCENT METHOD FOR SEQUENCE EMBEDDINGS

Gradient Descent Optimization

function GRADIENTDESCENT($h, nn_{indices}, nn_{dists}$) ▷ embeddings, nn ids, nn dists
    M = number of samples
    loss $= \sum_{m=1}^{M}$ computeLoss($m, h, h, nn_{indices}, nn_{dists}$) / M ;
    for epoch in ∞ **do** ▷ loop through epochs until breaking condition
        for i in shuffle(1...M) **do**
            sampleUpdate($i, h, nn_{indices}, nn_{dists}$)
        end for
        loss = lossUpdated
        lossUpdated $= \sum_{m=1}^{M}$ computeLoss($m, h, h, nn_{indices}, nn_{dists}$) / M ;
        if loss - lossUpdated $<$ .001 **break**
    end for
end function

subalgorithm Embeddings h update using sample i

function SAMPLEUPDATE($i, h, nn_{indices}, nn_{dists}$) ▷ $nn_{indices}, nn_{dists}$ lookup
    K = number of nearest neighbors
    $h_i \equiv h[i]$ ▷ embedding for i
    ϵ =1.0e-6 , λ =1.0e-2 ▷ regularization and update rate
    for k in 1...K **do**
        $h_{nn} \equiv h[nn_{indices}[k]]$
        $\delta \equiv d[k]$
        $D = \sqrt{(h_i - h_{nn}) \cdot (h_i - h_{nn})}$ ▷ standard euclidean distance between vectors
        $\Delta = \lambda(\delta - D) / \left(\delta^2(D + \epsilon)\right)$
        for j in 1...10 **do**
            $h_i[j]$+=$\Delta \cdot (h_i[j] - h_{nn}[j])$
        end for
    end for
end function

subalgorithm Loss for sample i

function COMPUTELOSS($i, h, nn_{indices}, nn_{dists}$) ▷ sample id, embeddings, nn ids, nn dists
    K =length($nn_{indices}$) =length(nn_{dists})
    $d \equiv nn_{dists}[i]$ ▷ d contains nearest neighbor distances for i
    $n \equiv nn_{indices}[i]$ ▷ n contains nearest neighbor indices for i
    $h_i \equiv h[i]$ ▷ embedding for i
    loss $= 0$
    for k in 1...K **do**
        $h_{nn} \equiv h[nn_{indices}[k]]$
        $D = \sqrt{(h_i - h_{nn}) \cdot (h_i - h_{nn})}$
        loss+= $\left|d[k] - D\right| / d[k]$
    end for
end function
