# Supplementary material for: Deep Learning Encoding for Rapid Sequence Identification on Microbiome Data
Source: Front Bioinform. 2022 Jun 24;2:871256. doi: 10.3389/fbinf.2022.871256 (PMC9580936; doi:10.3389/fbinf.2022.871256)
Supplement: Supplementary file 4 [file DataSheet1.ZIP › Borgman_et_al_SupplementaryMaterial/Supplementary Figure 1.pdf]

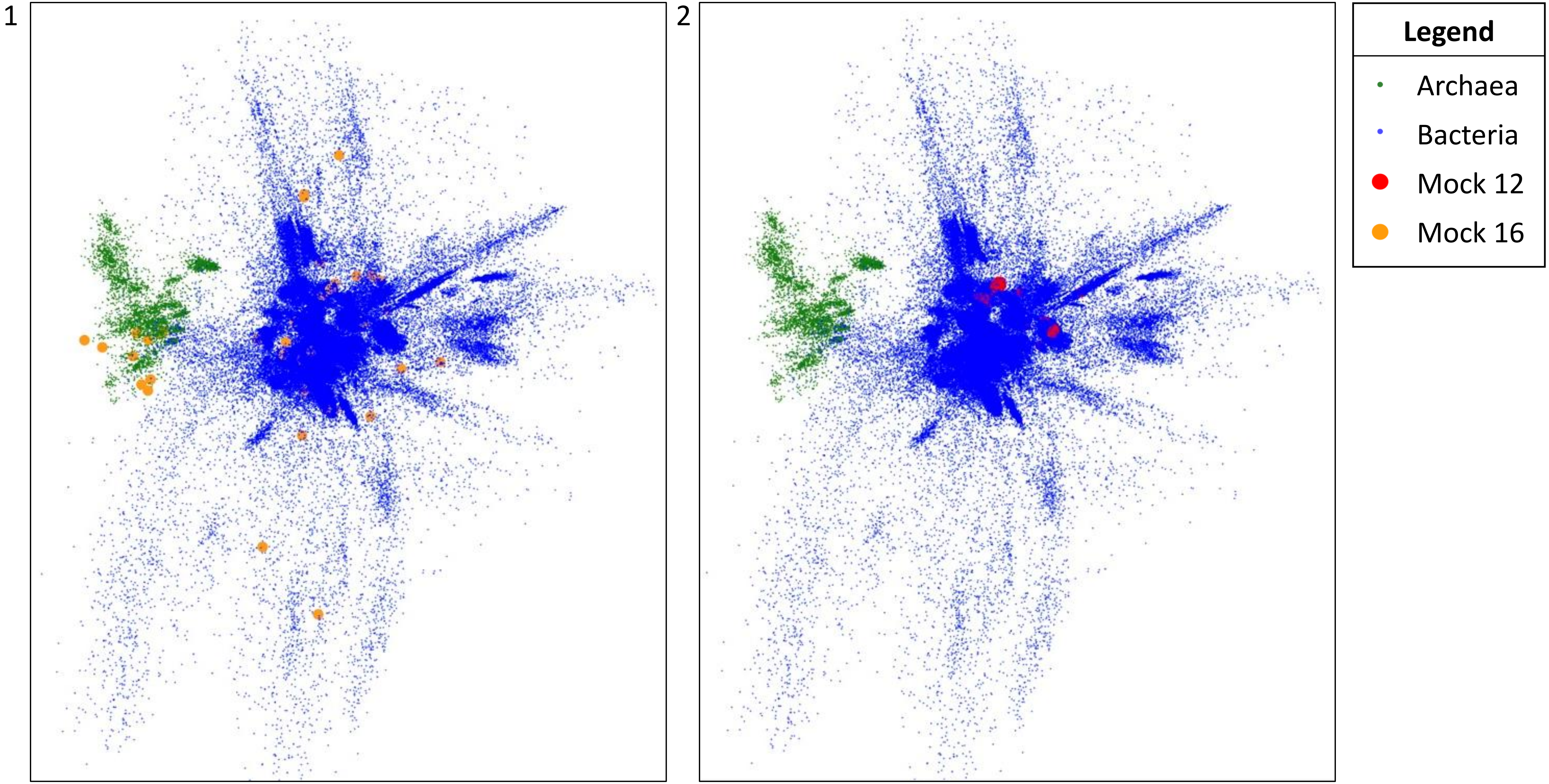

Supplementary Figure 1: DERSI analysis results in the 10-dimensional V4 latent space visualized in 3D using the first three Principal Components. Each dot represents a V4 sequence variant. All known bacterial V4s are shown as small blue dots and Archaeal V4s are shown as small green dots. In panel 1, the results of the V4 mappings by the trained convolutional neural network for the Mock 16 data are shown as large orange dots, while in panel 2, the V4 mappings for the less diverse Mock 12 data are shown as large red dots.
