# Supplementary material for: Deep Learning Encoding for Rapid Sequence Identification on Microbiome Data
Source: Front Bioinform. 2022 Jun 24;2:871256. doi: 10.3389/fbinf.2022.871256 (PMC9580936; doi:10.3389/fbinf.2022.871256)
Supplement: Supplementary file 4 [file DataSheet1.ZIP › Borgman_et_al_SupplementaryMaterial/Supplementary_Methods.pdf]

1 CHOOSING EMBEDDING AND ALIGNED SEQUENCE METRICS

For the phylogenetic embedding to be effective, the two distances d and D need be properly chosen. The euclidean distance, $d_L(a, b) = ||a - b||$ is used for the embedding space metric. The phylogenetic distance D should also be a true metric, i.e. satisfying the axioms:

$$D(x, x) \geq 0 \tag{1}$$

$$D(x, y) = 0 \iff x = y \tag{2}$$

$$D(x, y) = D(y, x) \tag{3}$$

$$D(x, y) \leq D(x, z) + D(z, y) \tag{4}$$

The last requirement is known as the triangle inequality. Without it, even the basic notion of a nearest-neighbor breaks down, since the ‘shortest’ distance between two points, as measured by the distance function, might be longer than another path taking a detour through a third point.

To formulate a valid and informative D, consider an aligned version of the training data set. Each sequence belongs to an N-dimensional product of the nucleotide character set, extended with padding and gap symbols: $\{A, C, G, T, \cdot, -\}^N$. Equivalently, we can regard each sequence s as the set of nucleotides $\{A_n, C_n, G_n, T_n\}^{|s|}$, where $|s|$ is the length of the unaligned sequence and where each nucleotide is indexed by $n \in \{1, \ldots, N\}$ designating its position in the global alignment. Using this representation, our phylogenetic distance is defined as the volume (or cardinality) of the symmetric difference between two sequences, $|p \triangle q|$. This obeys the axioms above , and has the intuitive interpretation of counting the number of nucleotide positions that are not shared between the two sequences. In set terminology, $\triangle$ is the union of the two sets, minus their intersection:

$$p \triangle q \equiv (p \cup q) \setminus (p \cap q) \tag{5}$$

$$|p \triangle q| = |p \cup q| - |p \cap q| \tag{6}$$

$$= |p| + |q| - 2|p \cap q| \tag{7}$$

where in the final line the substitution $|p \cup q| = |p| + |q| - |p \cap q|$ is made. Equation 7 is convenient for efficient linear algebraic computations and used for our implementation:

$$p \triangle q = |p| + |q| - 2|p \cap q| \tag{8}$$

$$= \mathbf{p} \cdot \mathbf{p} + \mathbf{q} \cdot \mathbf{q} - 2\, \mathbf{p} \cdot \mathbf{q} \tag{9}$$

Equation 9 assumes that each sequence s is represented as a binary vector $\mathbf{s} \in \{0, 1\}^{4N}$. $\mathbf{s}$ is formed as the sum over $|s|$ indicator binary vectors of dimension $4N$, each with a single non-zero entry indicating

presence of nucleotide character c at alignment position i,

$$\mathbf{s} \equiv \sum_{(i,c)\in s} (0, 0, 0, ...1_{4\cdot i+c}..., 0, 0), \tag{10}$$

$$i \in \{1, \ldots, N\} \tag{11}$$

$$c \in \{A = 0, C = 1, G = 2, T = 3\} \tag{12}$$

For a general vector space, Equation 9, as a squared euclidean distance, would fail to be a true metric, since it violates the triangular inequality. However, with specialized binary vectors indicating set membership, it simply expresses the volume of the symmetric difference and is therefore guaranteed to obey 4.
