# Supplementary material for: Deep Learning Encoding for Rapid Sequence Identification on Microbiome Data
Source: Front Bioinform. 2022 Jun 24;2:871256. doi: 10.3389/fbinf.2022.871256 (PMC9580936; doi:10.3389/fbinf.2022.871256)
Supplement: Supplementary file 4 [file DataSheet1.ZIP › Borgman_et_al_SupplementaryMaterial/Supplementary_Table_Descriptions_Final.docx]

Borgman et al. Supplementary Data

**Supplementary Table 1. Mock 16 16S gene - V4 Region List**

Supplementary Table 1 provides the data describing the genome of origin and copy number of each unique16S gene V4 region included in the input DNA used to create this data set. We chose this data set because of its complexity and significant phylogenetic breadth, as it contains 59 species, 10 of which are Archaea. Since the original publication of this data set, 5 of the genomes have had their names officially changed. Based on the actual sequence of the reads in the data set, one of the original organisms was misidentified.

Column A contains the genus and species names of all 59 input genomes, and if they were changed, then the original names are in Column E. Four pairs of names are on the same line in Column A as they contain identical V4 regions. This is also mentioned in Column E.

Column B contains the original whole genome DNA input percent for each species in the Mock 16 community.

Column C contains the 16S gene V4 region copy number in each genome or genome pair listed. The V4 copy number varies from 1-20 per unique V4 sequence, while 7 genomes have multiple sequence variants of the V4 region. Note that although the genomes were input at equivalent percentages, since the genomes have varied numbers of 16S copies, not all V4’s will be present in equivalent amounts.

Column D contains the actual input percent for each unique 16S gene V4 region input into the Mock 16 community adjusted for the copy numbers. This input percent was used to calculate the accuracy of each analysis tool.

Column E contains notes.

**Supplementary Table 2. Mock 16 DERSI, DADA2 and VSEARCH Count Data Output**

This table contains the detailed results of the abundance counts expected based on input compared to those obtained by each of the three algorithms on the Mock 16 data set.

Columns A-C contain the count data and Columns D-F contain the percent of total counts for each ASV or OTU created by DERSI (Columns A & D), DADA2 (Columns B & E) or VSEARCH (Columns C & F) for the analysis of the Mock 16 data set.

Column G contains the true input percent for each unique 16S gene V4 region identified (also see Supplementary Table 1, Column D).

Column H contains the percent identity of the identified 16S gene V4 region to the closest known genome listed in Column I.

The sequence of the most abundant read in each ASV or OTU is in Column J.

The low read count of many of the Archaeal genomes and the lack of reads for *Rhodospirillum rubrum* is most likely explained by the primer set used, as discussed in (Allaband et al., 2019).

Allaband C, McDonald D, Vázquez-Baeza Y, Minich JJ, Tripathi A, Brenner DA, Loomba R, Smarr L, Sandborn WJ, Schnabl B, Dorrestein P, Zarrinpar A, Knight R. Microbiome 101: Studying, Analyzing, and Interpreting Gut Microbiome Data for Clinicians. Clin Gastroenterol Hepatol. 2019 Jan;17(2):218-230. doi: 10.1016/j.cgh.2018.09.017. Epub 2018 Sep 18. PMID: 30240894; PMCID: PMC6391518.

**Supplementary Table 3. Mock 12 16S gene - V4 Region List**

This table provides the data describing the genome of origin and copy number of each unique16S gene V4 region included in the input DNA used to create this data set.

Column A contains the genus and species names of all input genomes.

Column B contains the percent of total DNA input for that genome, which would also be the expected abundance in the output read count.

Column C contain the 16S gene V4 region copy number for each input genome if it could be found.

Some of the genomes included in the mock community do not yet have a complete genome deposited in GenBank and therefore the exact copy number unknown. In those cases, the copy number presented is an estimate based on the average of the copy numbers found in several closely related genomes. When the copy number is an estimate it is followed by a ‘?’.

**Supplementary Table 4. Mock 12 DERSI, DADA2 and VSEARCH Count Data Output**

This table contains the count data (Columns A-C) and percent of total counts (Columns D-F) for each ASV or OTU created by DERSI (Columns A & D), DADA2 (Columns B & E) or VSEARCH (Columns C & F) for the analysis of the Mock 12 data set.

Column G contains the true or estimated true input percent for each unique 16S gene V4 region identified (also see Supplementary Table 3, Column D).

Column H contains the percent identity of the identified 16S gene V4 region to the closest known genome listed in Column I.

The sequence of the most abundant read in each ASV or OTU is in Column J.
