# Supplementary material for: Deep Learning Encoding for Rapid Sequence Identification on Microbiome Data
Source: Front Bioinform. 2022 Jun 24;2:871256. doi: 10.3389/fbinf.2022.871256 (PMC9580936; doi:10.3389/fbinf.2022.871256)
Supplement: Supplementary file 5 [file DataSheet5.docx]

Borgman et al. Supplementary Data

**Supplementary Table 1. Mock 16 16S gene - V4 Region List**

Supplementary Table 1 presents a row for each unique 16S V4 sequence in the input DNA used to create the Mock 16 community. It provides the detailed data describing the genome of origin and copy number of each unique 16S V4 region included in this data set. This presents the basis for and calculation of both the expected numbers of unique ASVs that should be detected for this data set, and the anticipated abundance for each of those ASVs.

We chose this data set because of its complexity and significant phylogenetic breadth, as it contains 59 species, 10 of which are Archaea. Since the original publication of this data set, 5 of the genomes have had their names officially changed and we use the newer names here, while noting its prior nomenclature. In addition, based on the actual sequence of the reads in the data set, we conclude that one of the original organisms was misidentified and we have listed it under the corrected genus and species. Both of these types of name changes are noted in column E.

Here is the definition of each column:

Column A (labeled: Taxonomy) contains the current genus and species names of all 59 input genomes. If the names have been changed, then the original names are noted in Column E. Since each row of the table corresponds to a unique V4 sequence, when the V4 sequence is identical across more than one of the mock community species, they are combined into the same row. Column A therefore contains five rows with more than one species name. This is also mentioned in Column E.

Column B (labeled: whole genome input %) contains the percentage of the total whole genome DNA that was input for each species (or identical V4 species pair) to form the Mock 16 community. Note that the column total shown on row 65 is not quite 100% due to rounding error in Excel.

Column C (labeled: V4 copy #) contains the 16S gene V4 region copy number for each row. The number of identical copies within a genome of the same V4 sequence varies from 1-10, while 7 genomes have additional sequence variants of the V4 region. If a genome has multiple versions of the V4 region the additional versions and their copy numbers are on the row below the first row for that species, and there is a blank for the name in Column A since it represents the same species. Note that although the genomes were input at equivalent percentages, since the genomes do have varied numbers of 16S copies, sometimes with V4 sequence variation, not all V4’s will be present in equivalent amounts.

Column D (labeled: V4 Expected Abundance %) contains the actual input percent for each unique 16S gene V4 region input into the Mock 16 community adjusted for the copy numbers. This input percent was used to create expected abundances and thereby enable calculating the accuracy of the abundance estimates obtained in the benchmark of the microbiome tools.

Column E (labeled: Old Genome Name / Comments) contains notes

Column F (labeled: Row identified in Supplementary Table 2) is provided to enable cross referencing from this table to Supplementary Table 2. It denotes the row in Supplementary Table 2 that shows the results for the identification and abundance of the same V4 by each microbiome algorithm. Note that 3 of the input V4s did not produce any reads in the data set~~.~~

**Supplementary Table 2. Mock 16 DERSI, DADA2, VSEARCH and UNOISE3 Count Data Output**

This table contains the detailed results of the abundance counts expected based on input compared to those obtained by each of the four algorithms on the Mock 16 data set. A count refers to a single sequence read from the input 16S V4 sequence data. Each row represents a unique V4 ASV or OTU identified by at least one of the microbiome analysis tools. Three groups of V4 sequences are presented, separated by a blank row. Rows 2-61 represent V4 sequences expected based on the input to the mock community. Rows 63-84 represent V4 sequences that were not intentionally input but appear to represent probable contaminants with exact matches to a known sequence. Rows 86-95 represent V4 sequences with no exact known matches whose origin is unclear.

Columns A-D contain the count data and Columns F-I contain the percent of total counts for each ASV or OTU created by DERSI (Columns A & F), DADA2 (Columns B & G), VSEARCH (Columns C & H), or UNOISE (Columns D & I) for the analysis of the Mock 16 data set.

Column K contains the expected input percent for each unique 16S gene V4 region identified (also see Supplementary Table 1, Column D).

Column L contains the percent identity of the identified 16S gene V4 region to the closest known genome listed in Column M.

Column M contains the name of the closest identified genome for that V4.

The sequence of the most abundant read in each ASV or OTU is given in Column N.

The low read count of many of the Archaeal genomes and the lack of reads for *Rhodospirillum rubrum* is most likely explained by the primer set used, as discussed in (Allaband et al., 2019).

Allaband C, McDonald D, Vázquez-Baeza Y, Minich JJ, Tripathi A, Brenner DA, Loomba R, Smarr L, Sandborn WJ, Schnabl B, Dorrestein P, Zarrinpar A, Knight R. Microbiome 101: Studying, Analyzing, and Interpreting Gut Microbiome Data for Clinicians. Clin Gastroenterol Hepatol. 2019 Jan;17(2):218-230. doi: 10.1016/j.cgh.2018.09.017. Epub 2018 Sep 18. PMID: 30240894; PMCID: PMC6391518.

**Supplementary Table 3. Mock 12 16S gene - V4 Region List**

Supplementary Table 3 presents a row for each unique 16S V4 sequence in the input DNA used to create the Mock 12 community. It provides the detailed data describing the genome of origin, and the copy number of each unique16S gene V4 region included in the input DNA used to create this Mock 12 data set. This presents the basis for and calculation of both the expected numbers of unique ASVs that should be detected for this data set and the anticipated abundance for each of those ASVs. Column A (labeled: Taxonomy) contains the genus and species names of all input genomes subdivided into the 6 input abundance levels.

Column B (labeled: whole genome input %) contains the percentage of the total whole genome DNA that was input for each species to form the Mock 12 community.

Column C (labeled: V4 copy #) contains the 16S gene V4 region copy number for each input genome if it is known. Some of the genomes included in the mock community do not yet have a complete genome deposited in GenBank and therefore the exact copy number unknown. In those cases, the copy number presented is an estimate based on the average of the copy numbers found in several closely related genomes. When the copy number is estimated it is followed by a ‘?’ and noted in Column E.

If a genome has multiple versions of the V4 region the additional versions and their copy numbers are on the rows below the first row for that species, and there is a blank for the name in Column A since it represents the same species. Note that although the genomes were input at specific percentages, since the genomes do have varied numbers of 16S copies, not all V4’s will be present in the originally intended amounts.

Column D (labeled V4 Expected Abundance %) contains the actual input percent for each unique 16S gene V4 region input into the Mock 12 community adjusted for the copy numbers. This is calculated as the input % times the V4 copy number divided by the average copy number for that Level of input abundance.

Column E (labeled Comments) contains any notes or comments relevant to this V4 row.

Column F (labeled: Row identified in Supplementary Table 4) is provided to enable cross referencing from this table to Supplementary Table 4. It denotes the row in Supplementary Table 4 that shows the results for the identification and abundance of the same V4 by each microbiome algorithm. Note that if this column is blank for a specific V4 row, the data set did not contain any reads for that V4 region.

**Supplementary Table 4. Mock 12 DERSI, DADA2, VSEARCH and UNOISE Count Data Output**

This table contains the detailed results of the abundance counts expected based on input compared to those obtained by each of the four algorithms on the Mock 12 data set. A count refers to a single sequence read from the input 16S V4 sequence data. Each row represents a unique V4 ASV or OTU identified by at least one of the microbiome analysis tools.

For each abundance level that was input, the table shows a set rows that represent V4 sequences expected based on the input to the mock community, then sequences that were not intentionally input but appear to represent contaminants with exact matches to a known sequence and finally sequences with no exact match whose origin is unclear.

Column A contains notes and subdivides the genomes into the 6 input abundance levels.

Columns B-E contains the count data and Columns G-J contain percent of total counts for each ASV or OTU created by DERSI (Columns B & G), DADA2 (Columns C & H), VSEARCH (Columns D & I) and UNOISE (Columns E & J) for the analysis of the Mock 12 data set.

Column L contains the expected percent for each unique input 16S gene V4 region identified (also see Supplementary Table 3, Column D).

Column M contains the percent identity of the identified 16S gene V4 region to the closest known genome listed in Column N.

Column N contains either the name of the genome identified from the mock community input or the closest genome identified.

Column O contains the sequence of the most abundant read in each row.

**Supplementary Table 5. Mock 23 16S gene - V4 Region List**

Supplementary Table 5 presents a row for each unique 16S V4 sequence in the input DNA used to create the Mock 23 community. It provides the detailed data describing the genome of origin, and the copy number of each unique16S gene V4 region included in the input DNA used to create this data set. This presents the basis for and calculation of both the expected numbers of unique ASVs that should be detected for this data set and the anticipated abundance for each of those ASVs.

Column A (labeled: Taxonomy) contains the genus and species names of all input genomes subdivided into the 4 input abundance levels.

Column B (labeled: whole genome input %) contains the percentage of the total whole genome DNA that was input for each species to form the Mock 23 community.

Column C (labeled: V4 copy #) contains the 16S gene V4 region copy number for each input genome.

If a genome has multiple versions of the V4 region the additional versions and their copy numbers are on the rows below the first row for that species, and there is a blank for the name in Column A since it represents the same species. Note that although the genomes were input at specific percentages, since the genomes do have varied numbers of 16S copies, not all V4’s will be present in the originally intended amounts.

Column D (labeled V4 Expected Abundance %) contains the actual input percent for each unique 16S gene V4 region input into the Mock 12 community adjusted for the copy numbers. This is calculated as the input % times the V4 copy number divided by the average copy number for that Level of input abundance.

Column E (labeled Comments) contains any notes or comments relevant to this V4 row.

Column F (labeled: Row identified in Supplementary Table 6) is provided to enable cross-referencing from this table to Supplementary Table 6. It denotes the row in Supplementary Table 6 that shows the results for the identification and abundance of the same V4 by each microbiome algorithm. Note that if this column is blank for a specific V4 row, the data set did not contain any reads for that V4 region.

**Supplementary Table 6. Mock 23 DERSI, DADA2, VSEARCH and UNOISE Count Data Output**

This table contains the detailed results of the abundance counts expected based on input compared to those obtained by each of the four algorithms on the Mock 23 data set. A count refers to a single sequence read from the input 16S V4 sequence data. Each row represents a unique V4 ASV or OTU identified by at least one of the microbiome analysis tools.

For each abundance level that was input, the table shows a set rows that represent V4 sequences expected based on the input to the mock community, then sequences that were not intentionally input but appear to represent contaminants with exact matches to a known sequence and finally sequences with no exact match whose origin is unclear.

Column A contains notes and subdivides the genomes into the 4 input abundance levels.

Columns B-E contains the count data and Columns G-J contain percent of total counts for each ASV or OTU created by DERSI (Columns B & G), DADA2 (Columns C & H), VSEARCH (Columns D & I) and UNOISE (Columns E & J) for the analysis of the Mock 23 data set.

Column L contains the expected percent for each unique input 16S gene V4 region identified (also see Supplementary Table 5, Column D).

Column M contains the percent identity of the identified 16S gene V4 region to the closest known genome listed in Column N.

Column N contains either the name of the genome identified from the mock community input or the closest genome identified.

Column O contains the sequence of the most abundant read in each row.
